# SMIFH2 has effects on Formins and p53 that perturb the cell cytoskeleton

**DOI:** 10.1038/srep09802

**Published:** 2015-04-30

**Authors:** Tadamoto Isogai, Rob van der Kammen, Metello Innocenti

**Affiliations:** 1 Division of Molecular Genetics, The Netherlands Cancer Institute, Amsterdam, The Netherlands

## Abstract

Formin proteins are key regulators of the cytoskeleton involved in developmental and homeostatic programs, and human disease. For these reasons, small molecules interfering with Formins’ activity have gained increasing attention. Among them, small molecule inhibitor of Formin Homology 2 domains (SMIFH2) is often used as a pharmacological Formin blocker. Although SMIFH2 inhibits actin polymerization by Formins and affects the actin cytoskeleton, its cellular mechanism of action and target specificity remain unclear.

Here we show that SMIFH2 induces remodelling of actin filaments, microtubules and the Golgi complex as a result of its effects on Formins and p53.

We found that SMIFH2 triggers alternated depolymerization-repolymerization cycles of actin and tubulin, increases cell migration, causes scattering of the Golgi complex, and also cytotoxicity at high dose. Moreover, SMIFH2 reduces expression and activity of p53 through a post-transcriptional, proteasome-independent mechanism that influences remodelling of the cytoskeleton.

As the action of SMIFH2 may go beyond Formin inhibition, only short-term and low-dose SMIFH2 treatments minimize confounding effects induced by loss of p53 and cytotoxicity.

Actin and microtubule networks are actively remodelled in response to external and internal signals to facilitate proper execution of a large number of cellular processes, such as formation of membrane protrusions and invaginations for cell movement and endocytosis, segregation of chromosomes during mitosis, abscission of daughter cells during cytokinesis, establishment of cell polarity, vesicle and organelle movement^1^,^2^. Not surprisingly, genetic studies indicate that actin and tubulin are instrumental for development and tissue homeostasis in mice^3^,^4^,^5^,^6^,^7^,^8^,^9^,^10^,^11^ and compelling evidence links them to human pathologies^12^,^13^,^14^.

Actin and tubulin exist as monomers that can reversibly form polymeric structures referred to as Filamentous actin (F-actin) and microtubule, respectively. Transition between these alternative states is controlled by numerous actin- and microtubule-binding proteins. Among these, tumour associated protein p53 and Formin-family of proteins (Formins) can modulate both actin and microtubule networks.

p53 is a transcription factor commonly deleted or mutated in several types of cancer^15^,^16^ that, besides controlling apoptosis and cell-cycle arrest in response to a variety of physiological and noxious signals^15^, also affects cell migration and invasion. Wild-type p53 modulates the activity of Rho-GTPases and the actin cytoskeleton to suppress directed cell migration and invasion^17^,^18^,^19^,^20^,^21^,^22^. Moreover, some p53 mutants possess transcription-independent pro-invasive functions^16^,^23^,^24^. As actin and microtubule dynamics control p53 activity^25^,^26^,^27^, regulation of p53 and cytoskeleton are interlaced with each other.

Formins are an evolutionary conserved protein family that binds monomeric actin and polymerizes it into filamentous actin through their Formin Homology 2 (FH2) domain. Recent studies have indicated that several Formins (mDia1, mDia2, mDia3, INF1, FMN1, FMN2 and Cappuccino) are also able to bind microtubules and increase their stability^28^,^29^,^30^,^31^,^32^,^33^,^34^,^35^. *Vice versa*, actin and tubulin can modulate Formins’ properties^29^. These observations suggest that Formin regulation, microtubule and actin dynamics are mutually interlinked. Consistently, Formins dictate proper execution of several cellular processes relying on the cytoskeleton^36^. Finally, increasing evidence links Formins to cancer and other diseases, suggesting that they may be potential new candidates for targeted therapy^37^,^38^.

Small molecule inhibitor of Formin Homology 2 domains (SMIFH2) is a cell-permeable compound that inhibits Formin-dependent actin polymerization by targeting the FH2 domain^39^. This property qualifies SMIFH2 as a general Formin inhibitor that may block the activity of all 15 Formin-family proteins. SMIFH2 has been used in many laboratories to explore Formins’ function in a wide range of biological contexts and processes^40^,^41^,^42^,^43^,^44^,^45^,^46^,^47^,^48^,^49^,^50^,^51^,^52^,^53^,^54^,^55^,^56^,^57^,^58^,^59^,^60^,^61^,^62^,^63^,^64^,^65^. Yet, observed effects, applied concentrations and treatment duration are quite variable and highlight lack of a thorough characterization of SMIFH2^40^,^41^,^42^,^43^,^44^,^45^,^46^,^47^,^48^,^49^,^50^,^51^,^52^,^53^,^54^,^55^,^56^,^57^,^58^,^59^,^60^,^61^,^62^,^63^,^64^,^65^.

In this study we find that SMIFH2 causes alternated depolymerization-repolymerization cycles of actin and microtubules, as well as scattering of the Golgi complex, whose onset correlates with post-transcriptional downregulation of Formin mDia2 and p53. While SMIFH2 attenuates p53-transcriptional activity independently of mDia2, it may induce cytoskeletal remodelling as a result of both Formin and p53 inhibition.

As we also report that SMIFH2 triggers cytotoxicity at high doses, we recommend current and future SMIFH2 users to employ short incubation (< 1 hour) and moderate concentrations (< 25 µM) to minimize confounding effects induced by loss of p53 and cytotoxicity. Under this provision, SMIFH2 represents a useful tool to study Formins both at the cellular and organismal levels.

## Results

### Single dose of SMIFH2 induces alternated depolymerization-repolymerization cycles of actin and tubulin

SMIFH2 is frequently exploited as pharmacological Formin inhibitor in loss-of-function studies. Consistent with Formins being key actin-regulatory proteins, many reports showed that SMIFH2 perturbs the actin cytoskeleton and gives rise to a constellation of actin-dependent phenotypes (Table 1). However, the broad range of employed concentrations and incubation times prevent comparative analyses and raises concerns about target specificity of SMIFH2 (Table 1). Despite Formins’ ability to regulate also microtubule dynamics, no information is available as to whether and how SMIFH2 affects microtubules. Together, these considerations show that a thorough and systematic characterization of SMIFH2 is missing.

We treated U2OS and HCT116 cells with SMIFH2 and monitored the remodelling of the actin cytoskeleton and microtubules over time using confocal microscopy. In order to compare different time points of treatment, we stained cells in parallel and used identical settings for image acquisition.

**Figure 1:**
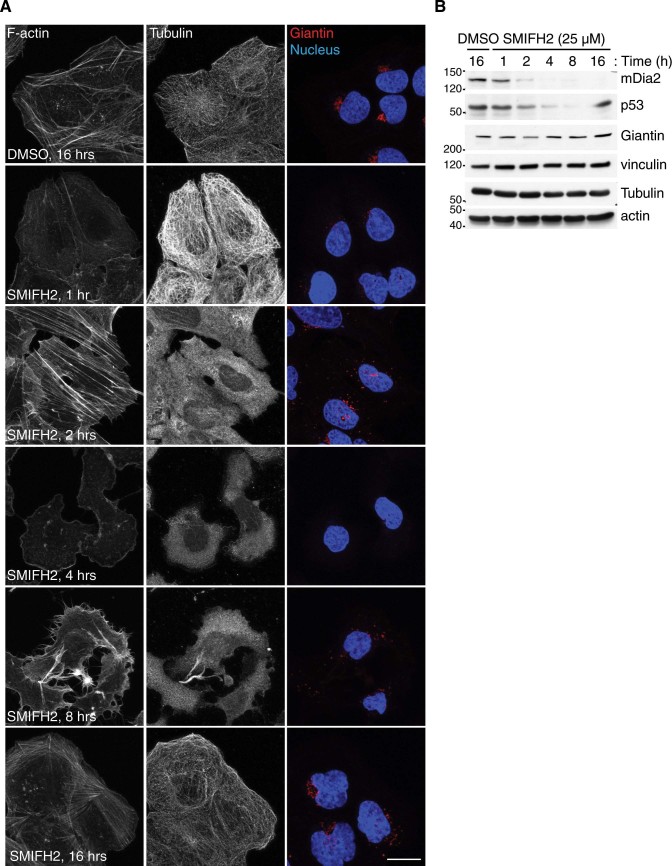
SMIFH2 affects the cytoskeleton and the Golgi complex of U2OS cells. (A) SMIFH2 induces dynamic cytoskeletal remodelling in U2OS cells. U2OS cells were treated with SMIFH2 or DMSO for the indicated time (Time, (h) = hour). Fixed cells were stained with anti-β-tubulin (Alexa-488) and anti-Giantin antibodies (Alexa-647; red in merge), TRITC-conjugated phalloidin to visualize F-actin, and DAPI to stain the nucleus (blue in merge). Representative maximal confocal projections are shown. Scale bar, 20 µm. (B) Downregulation of mDia2 and p53 by SMIFH2 temporally overlap. U2OS cells were treated with either DMSO or SMIFH2 in parallel as in (A). Total cell lysates were separated by SDS-PAGE and blotted with the indicated antibodies. Vinculin served as loading control. Gels were run under the same experimental conditions and blots were cropped for final display.

**Figure 2:**
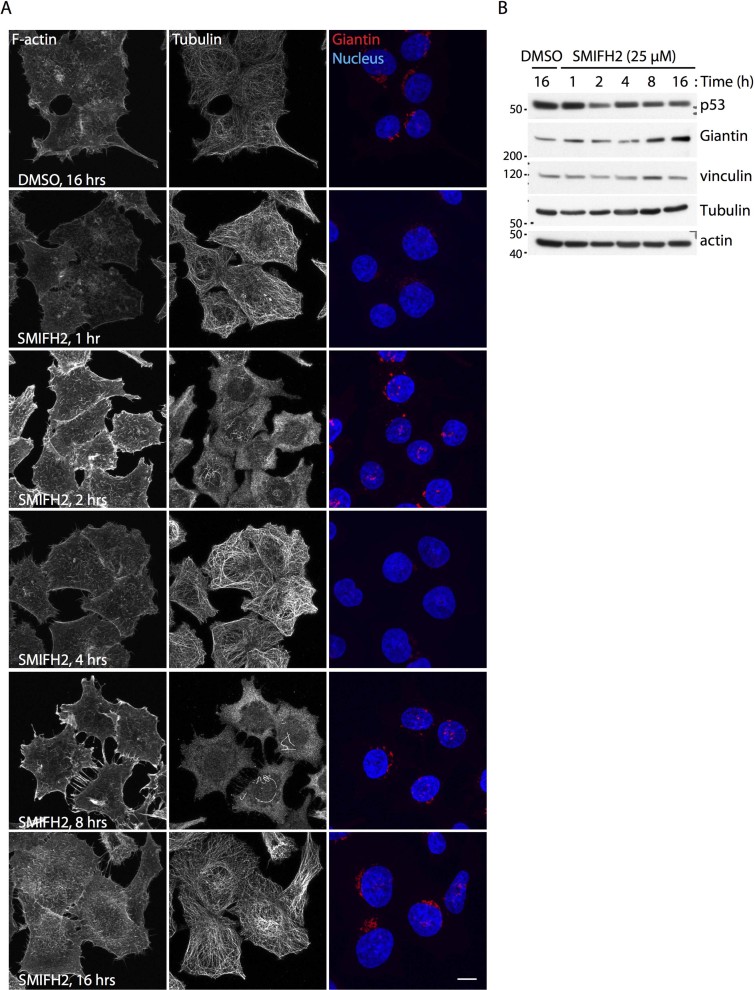
SMIFH2 affects the cytoskeleton and the Golgi complex of HCT116 cells. (A) SMIFH2 induces dynamic cytoskeletal remodelling in wild-type HCT116 cells. Wild-type HCT116 cells were treated as in Fig. 1A. Fixed cells were stained for F-actin (TRITC-Phalloidin), β-tubulin (Alexa-488), Giantin (Alexa-647; red in merge) and the nucleus (DAPI; blue in merge). Representative maximal confocal projections are shown. Scale bar, 10 µm. (B) SMIFH2 decreases p53 levels in HCT116 cells. Wild-type HCT116 cells were treated with SMIFH2 or DMSO for the indicated time (Time, (h) = hour). Total cell lysates were separated by SDS-PAGE and blotted with the indicated antibodies. Vinculin served as loading control. Gels were run under the same experimental conditions and blots were cropped for final display.

Control U2OS and HCT116 cells showed well-organized actin and microtubule networks (Fig. 1A and 2A). Cells incubated for one hour with SMIFH2 displayed reduced phalloidin signal accompanied by an increase in that of tubulin (Fig. 1A and 2A). As actin and tubulin protein levels did not vary in either U2OS or HCT116 cells (Fig. 1B and 2B), there appears to be a drop in cellular filamentous actin and a concomitant increase in polymeric tubulin. After two hours of treatment, stress fibres and apical filopodia-like protrusions formed in U2OS and HCT116 cells, respectively (Fig. 1A and 2A). Notably, the actin cytoskeleton of U2OS cells consisted primarily of lamellipodia and shaped into filopodia-like protrusions after four and eight hours of treatment with SMIFH2, respectively (Fig. 1A). Although HCT116 cells did not show lamellipodia at four hours, they formed basal filopodia-like protrusions after eight hours of treatment with SMIFH2 (Fig. 2A).

Two hours after addition of SMIFH2, microtubules were barely detectable in both U2OS and HCT116 cells (Fig. 1A and 2A, respectively). Lack of microtubules persisted up to the eight-hour time point in U2OS cells (Fig. 1A). Conversely, HCT116 cells showed some microtubules resistant to SMIFH2-induced depolymerization and also underwent a full repolymerization-depolymerization cycle between four and eight hours (Fig. 2A). Both U2OS and HCT116 cells reacquired a morphologically normal cytoskeleton when exposed to SMIFH2 for more than sixteen hours (Fig. 1A, 2A and not shown). The finding that SMIFH2 induces alternated depolymerization-repolymerization cycles of actin and microtubules was confirmed by live-cell imaging (Supplementary Movie S1) and has three possible explanations: *i)* SMIFH2 undergoes intracellular breakdown and/or inactivation, which progressively lowers SMIFH2’s active concentration below that required for full inhibition of all Formins, or *ii)* SMIFH2 has different and Formin-specific inhibitory effects on the actin and microtubule-regulatory activities, or *iii)* it has additional unknown targets. To gain insight into this issue, we treated U2OS cells with SMIFH2 and, every two hours, replaced the medium containing the inhibitor. Under this regimen, SMIFH2 addition caused a progressive and persistent depolymerization of both the actin cytoskeleton and microtubules (Supplementary Fig. S1A). As the SMIFH2-containing medium was prepared at the beginning of the time course, these results suggest that the depolymerization-repolymerization cycles of actin and microtubules are due to intracellular SMIFH2 breakdown/inactivation rather than its instability.

### SMIFH2 increases cell migration and prevents mitosis

**Figure 3:**
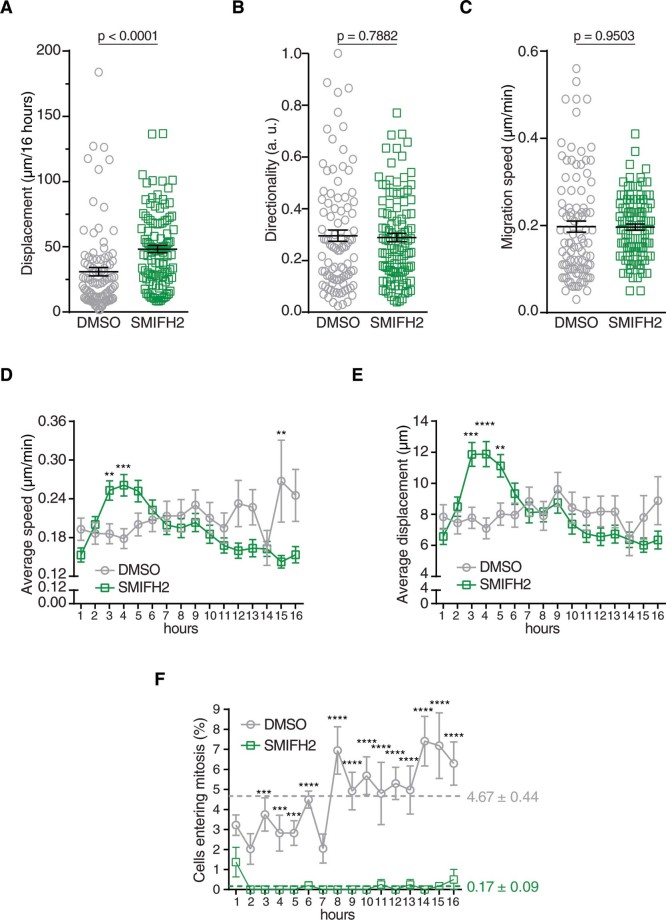
SMIFH2 affects migration and cell division of U2OS cells. (A-C) SMIFH2 increases cell movement. DMSO- or SMIFH2-treated cells were manually tracked and analysed for (A) net displacement, (B) directionality during movement and (C) migration speed as described in the Methods. Averages and SEM are indicated with black lines (Unpaired t-test; *n* = 96-106 cells from at least two independent experiments). (D) SMIFH2 increases migration speed during the first few hours of treatment. Average migration speed of cells tracked in (C) was plotted per every hour. (Unpaired t-test; ** = *p* < 0.01; *** = *p* < 0.001; *n* = 96-106 cells from at least two independent experiments). (E) SMIFH2-treated cells move farther during the first few hours of treatment. Displacement of cells was determined from cells tracked in (A). Data were plotted as in (D). (Unpaired t-test; ** = *p* < 0.01; *** = *p* < 0.001; **** = *p* < 0.0001; *n* = 96-106 cells from at least two independent experiments). (F) SMIFH2-treated cells show reduced mitotic entry. DMSO- and SMIFH2-treated cells entering in mitosis were detected as described in the Methods. Percentage of cells entering mitosis was plotted per every hour. Data represents average and SEM (n = 463-504 cells from at least two independent experiments). Unpaired t-test was employed to assess statistical significance (Unpaired t-test; *** = *p* < 0.001; **** = *p* < 0.0001). Colour-coded dashed lines highlight the average mitotic index over 16 hours.

The formation of SMIFH2-induced lamellipodia in U2OS cells prompted us to analyse cell migration. We manually tracked individual DMSO- or SMIFH2-treated U2OS cells and calculated total displacement, directionality and migration speed. SMIFH2-treated cells showed enhanced motility compared to DMSO-treated control cells, whereas speed and directionality did not significantly change (Fig. 3A-C, and Supplementary Movie S2). Interestingly, migration speed and displacement of SMIFH2-treated U2OS cells increased between three and five hours (Fig. 3D-E), temporally correlating with lamellipodium formation. As directionality was not concomitantly affected (data not shown), these data suggest that SMIFH2 affects cell motility by transiently modulating actin-based protrusions.

In-depth analysis of these time-lapse experiments evidenced that while the DMSO-treated cell population underwent mitosis, this was instead a rare event in the cells exposed to SMIFH2 (Fig. 3F and Supplementary Movie S2). These results suggest that SMIFH2 delays (or abrogates) cell division and are consistent with Formins being implicated in cytokinesis.

### SMIFH2 perturbs the architecture of the Golgi complex

The integrity of the Golgi apparatus strictly depends on microtubule^66^ and actin dynamics,^67^ INF2 and mDia1 Formins^68^,^69^. Thus, we stained the Golgi complex in control and SMIFH2-treated cells using Giantin, a *bona fide* marker for this organelle. The Golgi complex underwent a dramatic remodelling induced by SMIFH2 in both U2OS and HCT116 cells, as illustrated in Figure 1A and 2A, respectively. After one hour of treatment, the intensity of Giantin staining started to decrease and the Golgi showed a scattered morphology. At four hours, most of the Giantin signal disappeared, whereas protein levels remained stable (Fig. 1B and 2B). Longer treatment with SMIFH2 resulted in gradual recovery of the normal Golgi structure, which was restored at sixteen hours (Fig. 1A and 2A). We confirmed that SMIFH2 causes scattering of the Golgi complex using the *cis-*Golgi marker GM130 and the *trans-*Golgi marker TGN46 (Supplementary Fig. S2A). As U2OS and HCT116 cells express different Formins (Supplementary Fig S3A-B), this effect seems to support that SMIFH2 is a general Formin inhibitor^39^. Strikingly, SMIFH2 failed to cause disappearance of Giantin and only resulted in a moderate scattering of the Golgi complex in mouse embryo fibroblasts (MEFs) (Supplementary Fig. S4). Moreover, MEFs also started to die at 8 hours post addition of 25 μM SMIFH2 (Supplementary Fig. S4A). These discrepancies may be due to either inter-species differences (*H. sapiens vs. M. musculus*) or the nature of the analysed cells (cancer cells *vs.* immortalized cells). Nevertheless, MEFs showed alternated depolymerization-repolymerization of both actin and tubulin (Supplementary Fig. S4).

### SMIFH2 reduces p300, mDia2 and p53 levels in a proteasome-independent manner

**Figure 4:**
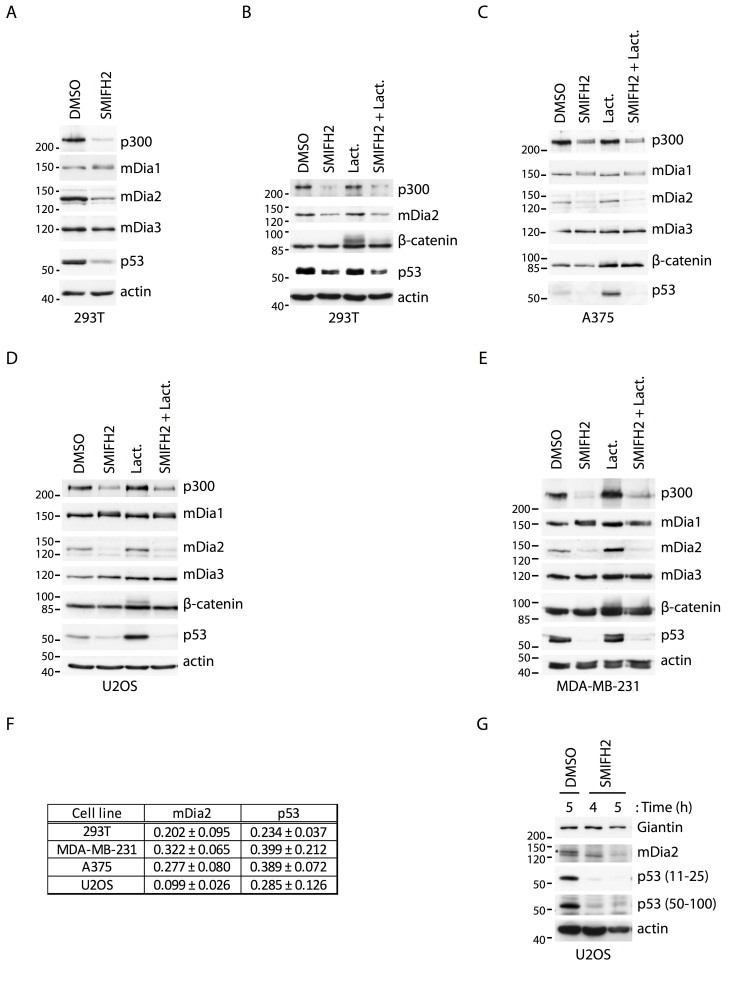
SMIFH2 downregulates mDia2, p53 and p300 protein levels. (A) SMIFH2 induces the downregulation of mDia2, p53 and p300 in 293T cells. 293T cells were treated with DMSO or SMIFH2 for five hours. Total cell lysates (30 μg) were separated by SDS-PAGE and immunoblotted with the indicated antibodies. One of two experiments that were performed with similar results is shown. (B-E) Proteasome inhibition fails to restore p53 levels during SMIFH2 treatment. (B) 293T, (C) A375, (D) U2OS, or (E) MDA-MB-231 cells were treated with DMSO (DMSO), SMIFH2 (SMIFH2), Lactacystin (Lact.), or SMIFH2 in combination with Lactacystin (SMIFH2 + Lact.) for five hours except for the A375 cells, which were treated for 2.5 hours. Total cell lysates (30 µg) were immunoblotted as indicated. One of two experiments that were performed with similar results is shown. (F) Quantification of mDia2 and p53 downregulation by SMIFH2. The expression of mDia2 and p53 were quantified by densitometric analyses of non-saturated films. For each of the indicated cell lines, intensities obtained in the SMIFH2- and DMSO-treated sample were normalized with respect to actin. Ratio between the normalized SMIFH2- and DMSO-treated intensities is represented as mean and SEM of at least three independent experiments. (G) Two different anti-p53 antibodies show that SMIFH2 reduces p53 levels. U2OS cells were treated with either DMSO or SMIFH2 for 4 and 5 hours (h = hours). Total cell lysates (30 μg) were separated by SDS-PAGE and immunoblotted with the indicated antibodies. Mouse monoclonal and rabbit polyclonal anti-p53 antibodies recognizing the region encompassing amino-acids 11-25 and 50-100 of p53, respectively, were used. One of two experiments that were performed with similar results is shown. A-E and G: Gels were run under the same experimental conditions and blots were cropped for final display.

Pilot dose-response experiments unmasked dramatic cytotoxic effects and cell death at SMIFH2 concentrations higher than 25 µM (Supplementary Movie S3), in agreement with previously reported observations^39^. As p53 is a master regulator of cellular apoptotic programs, we selected a few cells lines to cover all fundamental aspects of p53 biology. We employed 293T, A375, U2OS, MDA-MB-231 and HCT116 cells to take into account wild-type (293T, A375, U2OS, HCT116) *versus* mutant (MDA-MB-231) p53 status, high (293T, MDA-MB-231, HCT116) *versus* low (A375, U2OS) p53 expression and, proficient (293T, A375, U2OS, HCT116) *versus* deficient (MDA-MB-231) p53 transcriptional activity. Of note, these cell lines also have distinct Formin-expression landscapes (Supplementary Fig S3A-C and data not shown). We treated 293T, A375, U2OS and MDA-MB-231 cells with SMIFH2 and assessed the protein levels of p53 and its transcriptional co-activator p300. Expression of Diaphanous-related Formins mDia1, mDia2 and mDia3 was monitored as a control. Remarkably, we found that SMIFH2 reduced the protein level of mDia2, p53 and p300, while that of mDia1 and mDia3 were not affected (Fig. 4A-E). Notably, mDia1 showed a reproducible SDS-PAGE mobility shift suggesting a post-translational modification (Fig. 4A-E). Downregulation of p53 was clearly independent of p53 status, expression levels, and transcriptional activity and occurred in both immortalized (293T) and cancer (A375, U2OS, MDA-MB-231 and HCT116) cells of human origin (Fig. 4F). In agreement with this conclusion, SMIFH2 triggered downregulation of mDia2 and p53 also in MEFs (Supplementary Fig. S4B). Finally, we exploited two different anti-p53 antibodies to verify that SMIFH2 truly decreases p53 levels rather than causing post-translational modifications of p53 that prevent epitope recognition (Fig. 4G).

SMIFH2 inhibits Formins at the protein level and proteasome controls degradation of many cytosolic proteins, including p53 and mDia2^70^,^71^. Thus, we reasoned that SMIFH2 may promote proteasome-mediated disposal of mDia2, p300 and p53 and used the proteasome inhibitor Lactacystin to test this hypothesis. Lactacystin did not restore mDia2, p53 and p300 levels in SMIFH2-treated 293T, A375, U2OS and MDA-MB-231 cells, although it promoted the accumulation of β-catenin, a well-known substrate of the proteasome (Fig. 4B-E). Higher Lactacystin concentrations, longer treatment duration and different proteasome or protease inhibitors (MG-132 and ALLN, respectively) also failed to rescue mDia2, p53 and p300 levels (data not shown). The sum of these data strongly suggests that SMIFH2 induces proteasome-independent degradation of selective proteins, including p53, p300 and mDia2.

Next, we tested the possibility that SMIFH2 may alter abundance of messenger RNA of its targets. RT-qPCR on total mRNA isolated from SMIFH2-treated and DMSO-treated cells ruled out that SMIFH2 perturbs the mRNA levels of mDia2 and p53 (Supplementary Fig. S5A-B). These results support the notion that SMIFH2 acts at post-transcriptional level.

Collectively, these data show that downregulation of mDia2, p53 and p300 is a general and proteasome-independent effect of SMIFH2. Yet, the involvement of Formins in regulating gene expression post-transcriptionally remains unclear.

### SMIFH2 attenuates p53 transcriptional activity

**Figure 5:**
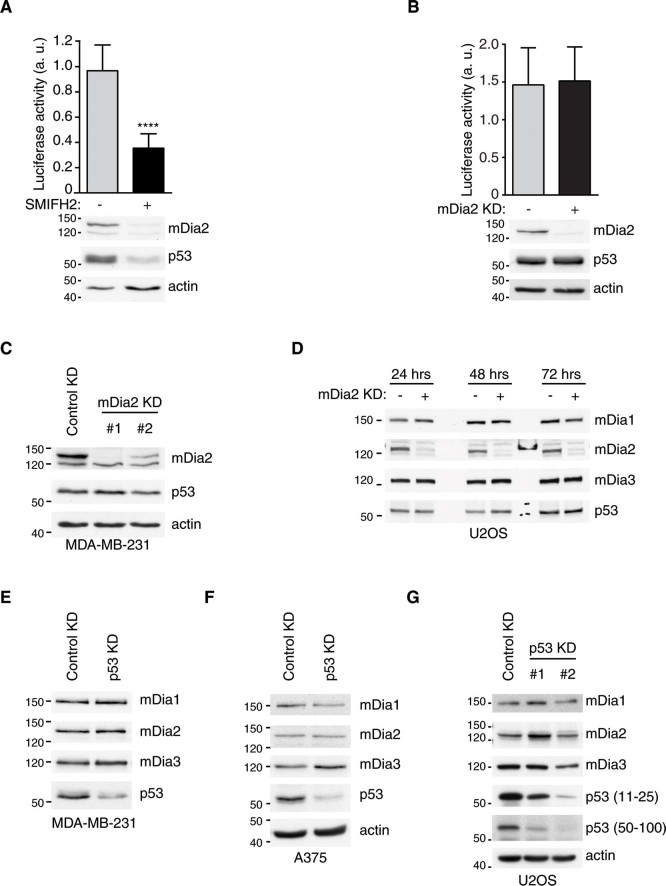
SMIFH2 attenuates p53 transcriptional activity independently of mDia2. (A) Pharmacological inhibition of Formins attenuates p53 transcriptional activity. 293T cells were transfected with the HDM2-luciferace reporter plasmid. Nineteen hours later, cells were treated with SMIFH2 (+) or DMSO (-) for five hours. Luciferase activity was measured as described in the Methods and plotted as mean ± s.d. of three independent experiments. Western Blotting confirmed downregulation of mDia2 and p53 protein expression. Unpaired t-test was used. *n* = 7 from three independent experiment; **** = *p* < 0.0001. (B) mDia2 knockdown does not affect p53 transcriptional activity. Luciferase activity was measured as in (A) in 293T cells with or without mDia2 knockdown. mDia2 knockdown was confirmed by Western Blotting. p53 levels remained equal after silencing of mDia2. (C-D) Knockdown of mDia2 does not alter p53 protein levels. Total cell lysates of (C) stable mDia2 knockdown MDA-MB-231 or (D) transient mDia2 knockdown U2OS cells processed at different time points were immunoblotted using the indicated antibodies. Empty lanes in (D) were loaded with reference protein markers. (E-F) Downregulation of p53 does not affect mDia2 protein levels. Total cell lysates of (E) transient p53 knockdown MDA-MB-231, (F) A375 cells and (G) stable p53 knockdown U2OS cells were immunoblotted with the indicated antibodies. A-G: Gels were run under the same experimental conditions and blots were cropped for final display.

p53 is a well-known transcription factor regulating expression of genes related to cell cycle, DNA damage repair and apoptosis^15^. Given that SMIFH2 reduces the expression of p53 and p300, we assessed the functional consequence of it on p53 transcriptional activity. To this end, we transfected 293T cells with a p53-responsive, HDM2 promoter-driven luciferase reporter plasmid, treated them with either SMIFH2 or DMSO and then measured the luciferase activity. These experiments revealed that addition of SMIFH2 attenuated p53-transcriptional activity compared to control samples (Fig. 5A).

Although SMIFH2 decreased both p53 and mDia2 expression, three observations suggest that reduced p53-transcriptional activity is independent of mDia2: knockdown of mDia2 did not affect *i)* either p53-transcriptional activity or *ii)* p53 protein levels (Fig. 5B-D), and *iii)* SMIFH2 reduced p53 levels also in HCT116 cells (Fig. 2B), where mDia2 is below detection limit (Supplementary Fig. S5C). Similarly, reduced mDia2 expression is independent of p53 levels as shown by the two following observations: *i)* silencing of p53 did not alter mDia2 protein levels (Fig. 5E-G), and *ii)* time-course experiments revealed the SMIFH2-induced dowregulation of mDia2 occurred with similar kinetics in both control and p53 knockdown U2OS cells (Supplementary Fig. S6A-B).

### SMIFH2-induced cytoskeletal remodelling and downregulation of p53 temporally overlap

Analysis of total cell extracts matching the time-course of Figures 1 and 2 revealed that p53 and mDia2 levels started to decline in U2OS one and two hour after SMIFH2 addition, respectively (Fig. 1B). In U2OS cells, expression of p53 was dramatically decreased at the eight-hour time point, whereas that of mDia2 was below detection limit already after four hours of treatment. Remarkably, normal p53 levels were fully rescued after sixteen hours, whilst mDia2 downregulation remained complete. Conversely, p53 remained low when SMIFH2 treatment involved replacing the inhibitor every two hours (Supplementary Fig. S1B). This observation corroborates the conclusion that SMIFH2 is broken down or inactivated within cells. In HCT116 cells, SMIFH2-induced downregulation of p53 became evident only at two hours of treatment and was less dramatic than in all other tested cell lines (Fig. 2B and Fig. 4A-E). Nevertheless, SMIFH2-induced remodelling of the cytoskeleton and p53 downregulation occurred as temporally correlated events also in HCT116 cells. It is worth noting that effectiveness of SMIFH2 treatment is not related to basal p53 expression as SMIFH2 strongly reduced p53 levels in 293T cells, which have more p53 than HCT116 cells (Fig. 4A-B and Supplementary Fig. S5C).

In addition to regulating gene transcription, p53’s action extends to the cytoskeleton^17^,^18^,^19^,^20^,^21^. As SMIFH2 reduced p53 expression and transcriptional activity, we wondered whether the effects of SMIFH2 on p53 could explain, at least partially, those on the cytoskeleton.

**Figure 6:**
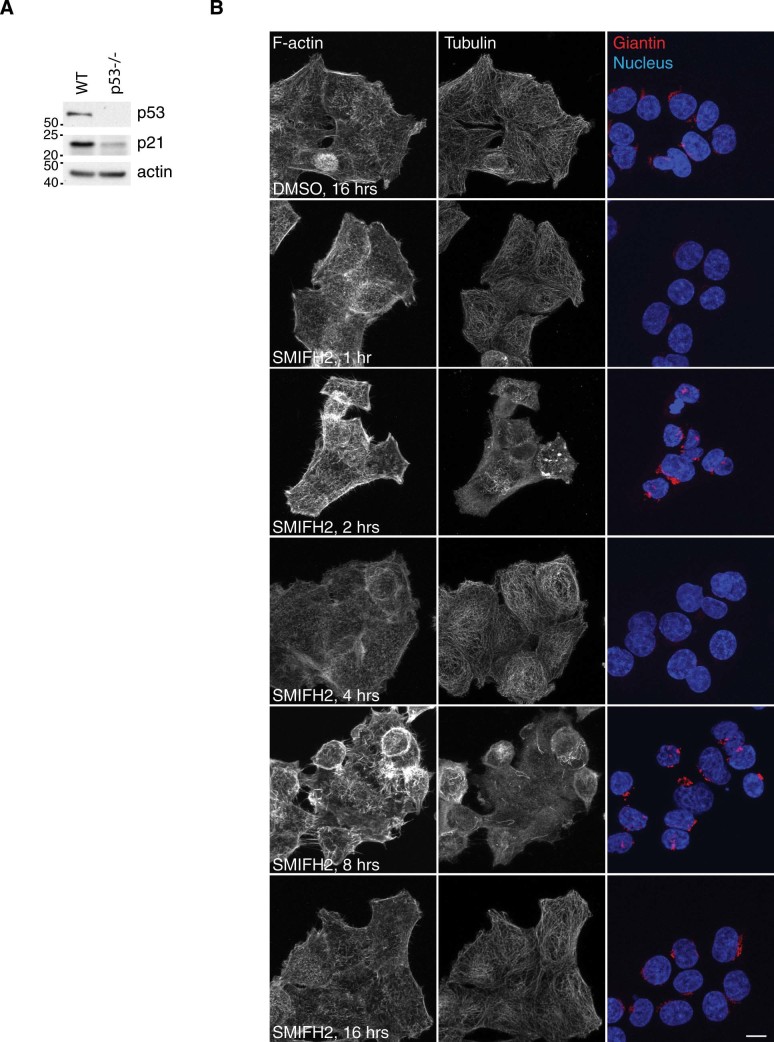
SMIFH2’s effects on the cytoskeleton are linked to p53 levels. (A) p53-/- HCT116 cells lack p53 and have decreased p21 levels. Total lysates were separated by SDS-PAGE and blotted with the indicated antibodies. Gels were run under the same experimental conditions and blots were cropped for final display. (B) SMIFH2’s effects on the cytoskeleton are linked to p53 levels. p53-/- HCT116 cells were treated and stained in parallel with those displayed in Figure 2A. Scale bar, 10 µm.

Side-to-side comparison of cytoskeletal remodelling evoked by SMIFH2 in wild-type and p53 -/- HCT116 cells showed that the decrease in phalloidin and concomitant increase in tubulin signals observed at the one-hour time point were linked to p53 expression (Fig. 2 and Fig. 6). Conversely, the two cell lines responded similarly to SMIFH2 from two hours onwards, namely since reduction of p53 levels in the p53 wild-type cells. Control and p53 knockdown U2OS cells showed that p53 expression levels regulate the actin cytoskeleton, whereas they do not affect either microtubules or Golgi organization (Supplementary Fig. S6A). Under growing conditions, we observed that the cortical actin cytoskeleton and stress fibres were more prominent in either p53 knockdown mass populations than in the corresponding control knockdown mass population (Supplementary Fig. S6A). Live-cell imaging of control and p53 knockdown U2OS cells expressing EGFP-LifeAct and mCherry-α-Tubulin unveiled that p53 is needed for SMIFH2 to induce the protrusion of lamellipodia (Supplementary Movie S4). Conversely, these experiments showed that SMIFH2’s effect on microtubule dynamics are independent of p53, at least in U2OS cells (Supplementary Movie S5).

As SMIFH2 reduces p53 expression and activity and p53 levels affect the cytoskeleton, the observed interplay between p53 and the cytoskeleton makes it difficult to ascribe any cytoskeletal effects induced by SMIFH2 treatments longer than one hour solely to Formin inhibition. In spite of that, SMIFH2’s effects on the Golgi complex did not seem to be modulated by p53 in either HCT116 or U2OS cells (Fig. 2 and Fig. 6 and Supplementary Movie S6).

## Discussion

In this study we found that the general Formin inhibitor SMIFH2 causes alternated depolymerization-repolymerization cycles of actin and microtubules, as well as scattering of the Golgi complex in human cells (Fig. 1 and Fig. 2). Surprisingly, SMIFH2 decreased the protein levels of p300, mDia2 and p53 without affecting the abundance of their messenger RNA. As proteasome inhibitors failed to restore the expression of p300, mDia2 and p53, we suggest that SMIFH2 regulates expression of these proteins post-transcriptionally and independently of the proteasome. Although it is formally possible that SMIFH2 might affect protein translation as such, we regard this possibility as unlikely since expression of other proteins (*e.g.* mDia1, mDia3, actin, tubulin and Giantin) remained unchanged (Fig. 4).

Consistent with the observed reduction in p53 and p300 levels, SMIFH2 attenuated p53 transcriptional activity independently of mDia2. The finding that expression of p53 and mDia2 were not interlinked confirms that there is no causal relationship between mDia2 and p53 regulation by SMIFH2 (Fig. 5).

Nonetheless, depolymerization of F-actin can activate p53 transcription^25^ and polymerization of G-actin inhibits p53 function^26^. Given that Formins are actin nucleators, it is reasonable to speculate that they directly or indirectly impact on the p53 pathway. Interestingly, a recent study implicated Formin FMN2 in stabilization of cyclin-dependent kinase inhibitor p21 during oncogene/stress-induced cell cycle arrest^72^.

Confocal imaging confirmed that SMIFH2 modulates the actin cytoskeleton as previously reported^39^, although observed phenotypes depend on both the cell line being tested and treatment duration (Fig. 1, Fig. 2 and Supplementary Fig. S4). We hypothesize that this is most likely due to Formins having cell-type-specific expression profiles (Supplementary Fig. S3) and different sensitivity to SMIFH2.

Under the condition that SMIFH2 has Formin-specific affinities and its active concentration drops relatively fast, the alternated depolymerization-repolymerization cycles of actin and microtubules triggered by SMIFH2 would agree with mDia1, mDia2, INF2, and possibly some other Formins, co-regulating the dynamics of F-actin and microtubules and either cytoskeletal network modulating Formins’ action on the other one^29^,^73^.

Most importantly, we discovered that SMIFH2 reduces both the expression levels and the transcriptional activity of p53 and that this property contributes to SMIFH2-induced cytoskeletal remodelling (Fig. 5, Fig. 2, Fig. 6 and Supplementary Fig. S6). The link between p53 and cytoskeletal remodelling is further strengthened by our re-analysis of a recent study identifying a set of 198 genes upregulated one hour after stabilization of p53 in HCT116 cells^74^. Among them, 77 were novel and previously uncharacterized p53 targets^74^. Using Ingenuity Pathway Analysis, we found that 43 out of the 192 mapped genes are early p53 target genes significantly associated with functions related to cellular movement (Table 2). In keeping with this notion, we noted that wild-type and p53 -/- HCT116 cells responded differently to SMIFH2 at early (< 1 hour), but not late time points. We established that p53 modulates the cellular responses to SMIFH2 both in HCT116 cells and U2OS cells by using syngenic wild-type and p53 knockout (or knockdown) cell lines (Supplementary Fig. S6 and Supplementary Movie S4).

Although SMIFH2 has been previously reported to exert anti-migratory effects,^39^ we found that it increased cell migration between three and five hours of treatment (Fig. 3). This discrepancy might be due to Rizvi and colleagues exposing NIH3T3 cells to a sub-lethal concentration of SMIFH2, NIH3T3 and U2OS cells differing in Formin-protein expression, or the experimental setup.

At any rate, SMIFH2 perturbs the architecture of the Golgi complex independently of p53 expression with a kinetics that differs from those of the actin and the microtubule networks (Fig. 1, Fig. 2, and Supplemental Movie S6). In this regard, loss of INF2 and activation of Rho-mDia1 pathway have been shown to result in partial dispersal of the Golgi complex in U2OS cells^68^,^69^. As virtually complete loss of visible Golgi structures occurs in U2OS and HCT116 cells treated with SMIFH2 (Fig. 1A and 2A), one or more Formin(s) may cooperate with INF2 in regulating Golgi architecture. The fact that perturbation of the Golgi complex and centrosomal microtubules are out of synchrony suggests that SMIFH2 might interfere with the dynamics of non-centrosomal microtubules nucleated at the Golgi, which are crucial for proper assembly and functionality of the Golgi complex^75^. Finally, why SMIFH2 does not elicit loss of the Golgi in MEFs warrants future investigation.

Remarkably, SMIFH2-treated cells recovered their original morphology after sixteen hours of treatment. Given that intracellular SMIFH2 decays in a few hours, this implies that inhibition of Formins by SMIFH2 is transient and reversible. In light of these considerations, it is notable that mDia2 remains fully silenced also when both the Golgi complex and the cytoskeleton have regained a normal morphology. Overall, our observations reinforce the idea that SMIFH2 may have different binding affinities for and ensuing inhibitory effects on Formins, and that the vast collection of perturbations induced by SMIFH2 (Table 1) results from different subsets of Formins being inhibited at any analysed time point.

High SMIFH2 concentration (50 µM) triggered rapid cell death in all tested cell lines (Supplementary Movie S3), consistent with previous observations and IC_50_ (IC_50 _ = 28.0 µM)^39^. As our data also show that this SMIFH2-induced event does not require p53 (Supplementary Movie S3), further studies should address the mode whereby SMIFH2 promotes cell death.

In summary, we showed that the general Formin inhibitor SMIFH2 has profound effects on F-actin, microtubules and integrity of the Golgi complex and influences important cellular processes, such as cell migration and cell division. Unexpectedly, we found that SMIFH2 also reduces the expression and the transcriptional activity of p53 and that this latter property may contribute to SMIFH2-induced cytoskeletal remodelling. Yet, it remains to be established whether p53 downregulation is caused by SMIFH2 inhibiting Formins or uncharacterized off-target effects of SMIFH2. As SMIFH2 affects both Formins and p53, we advise current and future users to administer SMIFH2 at moderate concentrations (< 25 µM) and employ short treatments (< 1 hour) to minimize confounding effects induced by loss of p53 and cytotoxicity. Under this provision, SMIFH2 remains a useful tool to study Formins both at the cellular and organismal levels.

## Methods

### Chemicals and Reagents

High-glucose DMEM supplemented with pyruvate and GlutaMax® was from Invitrogen. Dual-Luciferase® Reporter Assay System was from Promega. Lactacystin was from Cayman Chemicals and dissolved as 10 mM stock in DMSO and used at 10 µM. SMIFH2 was from Sigma-Aldrich and dissolved as 50 mM stock in DMSO and stored at –80°C in single-use aliquots. Thus, freeze-thaw cycles were limited to one since we noticed decreased activity upon additional cycles. SMIFH2 was used at 25 µM throughout this study, unless specified otherwise. Retroviral expression plasmid pMX-EGFP-LifeAct was generated by polymerase chain reaction and sequence verified. Retroviral expression plasmid pCX-mCherry-α-Tubulin was a kind gift from R. Wolthuis. pECFP-Golgi was from Clontech. pGL3-HDM2-luc reporter plasmid was a kind gift from R. Bernards. X-tremeGene9 was from Roche. All other reagents were purchased from Sigma-Aldrich.

### Antibodies

Antibodies were as follows: mouse monoclonal anti-β-actin (AC-15), anti-β-tubulin (clone B-5-1-2) and anti-vinculin (V9131) (Sigma-Aldrich), mouse monoclonal anti-mDia1 and anti-GM130 (BD Transduction Laboratories), rabbit polyclonal anti-p300 (C-20), mouse monoclonal anti-p53 (epitope 11-25; DO-1) (Santa Cruz Biotechnology), rabbit polyclonal anti-p53 (epitope 50-100; ab17990), mouse monoclonal anti-β-catenin (#2698) (Cell Signaling Technology), rabbit polyclonal anti-mDia3 (A300-079A) (Bethyl Laboratories), rabbit polyclonal anti-Giantin (ab24586). Rabbit polyclonal anti-TGN46 (Novus Biologicals). Anti-mDia2 sera were generated in house^76^.

### Cell Culture, Transfections and Knockdowns

293T, A375, U2OS, MEF and MDA-MB-231 cells were cultured in DMEM GlutaMax® (Invitrogen) supplemented with 10% FCS. HCT116 wild-type and p53-/- cells^77^ were a kind gift from B. Vogelstein and were cultured in DMEM/F12 (1:1) GlutaMax® supplemented with 10% FCS and 100 U/ml penicillin and 100 µg/ml streptomycin. mDia2 knockdown 293T cells were generated using lentiviral infection with pLL3.7^78^ harbouring a previously published sequence to silence mDia2^79^. mDia2 knockdown U2OS cells were generated with siRNA oligonucleotides as previously described^79^. Stable mDia2 knockdown MDA-MB-231 cells were obtained using the MISSION® TRC shRNA TRCN0000150903 (#1) and TRCN0000150850 (#2) (Sigma-Aldrich) and stable populations were selected with Puromycin. p53 knockdown A375 and MDA-MB-231 cells were obtained by retroviral infection with pRS-p53 and subsequent selection with Puromycin as previously published^80^. Stable p53 knockdown U2OS cells were obtained by lentiviral infection using MISSION® TRC shRNA TRCN0000003755 (#1) and TRCN0000003756 (#2) (Sigma-Aldrich) and selected with Puromycin for 1-2 days. EGFP-LifeAct and mCherry-α-Tubulin-expressing U2OS cells were generated by infection with retroviruses packaged in amphotropic Phoenix cells. pECFP-Golgi was transfected using X-tremeGene9 according to manufacturer’s instructions and cells were imaged 24-48 hours post-transfection.

Cells were lysed in JS lysis buffer (50 mM Hepes pH 7.5, 150 mM NaCl, 5 mM EGTA, 1.5 mM MgCl2, 1% glycerol and 1% Triton X-100) supplemented with fresh protease (10 µg/ml leupeptin and aprotinin, 1 mM Pefabloc) and phosphatase inhibitors (1 mM orthovanadate and 5 mM sodium fluoride).

### Total RNA Isolation and RT-qPCR Analyses

Total RNA from adherent cells was extracted using RNeasy Mini kit (Qiagen) according to manufacturer’s instructions. Complementary DNA synthesis was performed using 1-2 µg of mRNA with SuperScript-II® reverse transcriptase according to the manufacturer’s instructions (Invitrogen). Real-time qPCR reactions were set up using 5-10 ng of cDNA as a template and gene specific primers (200 nM) in a StepOnePlus^TM^ Real-Time PCR system (Applied Biosystems). All reactions produced single amplicons (100-200 bps), which allowed us to equate one threshold cycle difference. RT-qPCR primers are listed in Table 3 and have been previously validated^76^.

### Luciferase Activity Assay

293T cells (5 x 10^4^ cells/well) were plated in 24-well plates. One day after, cells were transfected with pGL3-HDM2-luc (100 ng) using calcium phosphate method. All experiments were carried out in triplicates and the Firefly Luciferase activity was measured 24 hours post-transfection with the Dual-Luciferase® Reporter Assay System (Promega) using an EnVision® Multilabel Reader (PerkinElmer). Initially, we added HDM2-Firefly luciferase-based reporter and *Renilla* luciferase-based co-reporter in a 1:10 ratio, which prevented *trans* effects between the two promoters (not shown). Incomplete quenching of the Firefly luciferase and low *Renilla* luciferase activity affected the normalization of the samples thereby causing misrepresentation of the effects (not shown). As total DNA amount was kept constant, we obtained very similar transfection efficiencies for different conditions and independent samples. Thus, the Luciferase activity was expressed as arbitrary units/µg of total proteins. Results are normalized against the control samples and represented as relative HDM2-promoter activities (mean ± s.d.), as obtained from three independent experiments carried out in technical triplicates.

### Immunofluorescence and Imaging

Cells were plated on gelatin-coated glass coverslips (#1.5) and fixed with 4% paraformaldehyde in PIPES buffer (80 mM PIPES pH 6.8, 5 mM EGTA, 2 mM MgCl2) for 10 minutes. Fixed cells were permeabilized in PBS containing 0.5% BSA (w/v) and 0.1% Triton-X100 (v/v) for 10 minutes, and stained with primary and secondary antibodies in blocking buffer (2.5% BSA (w/v) in PBS). Coverslips were mounted using Mowiol. Images were acquired on a CLSM Leica TCS SP5 operated with Leica Confocal Software (LAS-AF; Leica) and equipped with a HCX PL APO CS 63.0x (N.A. 1.40) oil objective. All channels were acquired sequentially.

Live cell confocal images were acquired on a CLSM Leica TCS SP5 equipped with a humidified climate chamber with 5% CO_2_ at 37**°**C. Single basal sections with a widened pinhole (1.5 Airy) were acquired every 20 minutes. Cells were left in the humidified climate chamber for at least one hour to obtain steady-state conditions prior to the beginning of each experiment. All channels were acquired sequentially. Images were corrected for photobleaching through estimation of the baseline intensity level by curve fitting the background intensities using “Exponential with offset” selection in ImageJ. These estimates were subsequently used for bleach correction in ImageJ with the simple ratio method^81^. Minor drift was corrected using the *TurboReg* and *StackReg* plugins in ImageJ^82^.

### Random Cell migration assay and quantification of cell motility and mitosis

Cells were plated on gelatin coated 6 or 12 wells plate and cells were allowed to adhere for at least sixteen hours. Experiments were performed in a humidified chamber with 5% CO_2_ at 37 **°**C in the presence of DMSO or SMIFH2. Cells were imaged every five minutes on a Zeiss Axio Observer Z1 microscope (Carl Zeiss) equipped with a LD Plan-Neofluar Ph2 20x (N.A. 0.40) objective, operated with Zeiss Microscope Software ZEN 2012. Individual cells were tracked using *Manual Tracking* plugin for ImageJ. Average distance, speed and directionality of movement were computed using the *Chemotaxis Tool* plugin for ImageJ provided by ibidi GmbH (http://www.ibidi.com).

Cells entering mitosis were scored manually and defined as follows: a cell entered mitosis when its flat and spread appearance changed to a round-up, yet adhesive state. Initial number of cells was counted manually in each field of observation and percentage of cells entering mitosis every hour was calculated using this initial number of cells as reference.

### Statistics

Statistical analyses were performed using GraphPad Prism version 6.01 for Windows (GraphPad Software, San Diego California USA, http://www.graphpad.com). Student’s unpaired t test was employed. *p* < 0.05 was considered statistically significant. ** = *p* < 0.01, *** = *p* < 0.001, **** = *p* < 0.0001.

## Supplementary Material

Supplementary Information Supplementary Information

Supplementary Information Supplementary Movie S1

Supplementary Information Supplementary Movie S2

Supplementary Information Supplementary Movie S3

Supplementary Information Supplementary Movie S4

Supplementary Information Supplementary Movie S5

Supplementary Information Supplementary Movie S6

## Figures and Tables

**Table 1:** List of studies using SMIFH2 (*as of September, 2014).*

Year	Study	Concentration used	Treatment duration	Purpose of use
2009	Rizvi et al., 2009^39^	5 - 30 µM	30 minutes up to 72 hours	Characterization of SMIFH2 in Fission yeast, NIH3T3 mouse fibroblasts and A549 human lung adenocarcinoma epithelial cells
2011	Poincloux et al., 2011^49^	25 µM	up to 4 hours	To study Formin-dependent invasive capacities of MDA-MB-231 human breast carcinoma cells
	Li et al., 2011^43^	100 nM	not specified	To study the relationship between Formin function and melanoblast motility
2012	Tang and Brieher, 2012^53^	not specified	30 minutes	To study the contribution of Formins during actin recovery in apical junctions of polarized MDCK cells after Latrunculin B treatment
	Wyse et al., 2012^56^	10 µM	~63 minutes	To study the role of mDia Formins in CXCL12-induced bleb formation in MDA-MB-231 human breast carcinoma cells
	Oakes et al., 2012^47^	10 – 15 µM	>4 hours	To confirm a previous study that formation of radial stress fibres formation in U2OS cells is Formin Dia1-dependent
	Miklavc et al., 2012^45^	25 µM	not specified	To show that actin coat formation on lamellar bodies in alveolar type II cells are Formin-dependent
	Chin et al., 2012^60^	10 µM	4 hours	To study the role of Formin-mediated cytoskeletal signaling in *Chlamydia* bacterial inclusion and extrusion from host cells (HeLa cells)
	Rosero et al., 2012^51^	5 - 30 µM	72 hours	To study the plant cell growth and morphogenesis of *Arabidopsis* plants
2013	Sandbo et al., 2013^52^	3 - 30 µM	30 minutes	To study myofibroblast differentation in human lung fibroblast cells
	Fritzsche et al., 2013^65^	40 µM	30 minutes	To analyse the contribution of Formin-mediated actin polymerization on actin cortex homeostasis
	Goldspink et al., 2013^40^	10 µM	40 minutes	To show that microtubule reorganization of EB-2 depleted ARPE-19 cells are restored to normal upon Formin inhibition
	Wilson et al., 2013^55^	40 µM	up to 490 seconds	To study the contribution of Formin-mediated actin polymerization at the leading edge of polarized HL60 neutrophil-like cells during 3D migration
	Rao et al., 2013^50^	30 µM	3 hours	As a negative control that activation of endogenous Diaphanous-related Formins by photoactivatable-DAD construct is indeed Formin dependent
	Aragona et al., 2013^58^	5, 15 or 30 µM	24 hours	To study the link between actin dynamics and Formin activity related to YAP/TAZ activity
	Yu et al., 2013^57^	50 µM	up to ~49 minutes	To study the requirement of Formin activity for podosome formation in RPTPalpha++ mouse embryonic fibroblasts
	Iskratsch et al., 2013^41^	5 - 10 µM	up to 40 minutes	To confirm the contribution of FHOD1 on cell spreading and adhesion maturation
	Luo et al., 2013^44^	20 - 40 uM	24 hours	To study the formation of actin nodes that mediate the formation of actin network within HeLa cells
	Murk et al., 2013^46^	75 µM	2 hours	To study the contribution of Formin on transition of stellate astrocytes to polygonal cells during Arp2/3 complex inhibition
	Buvall et al., 2013^59^	10 µM	90 minutes	To examine the Formin-dependency of stress fibre formation in podocytes
2014	Jennings et al., 2014^42^	10 µM	pretreatment not specified	To show that priming or degranulation of neutrophil is suppressed by Formins downstream of RhoA
	Pettee et al., 2014^48^	10 µM	48 hours	To confirm that organized ovarian spheroid formation of ES-2 cells is dependent on mDia2
	Tien and Chang, 2014^54^	10 - 100 µM	4 hours	To show the correlation between Dia1 inhibition and regulation of ERK activity in MDCK cells
	Beckham et al., 2014^61^	10 µM	4 hours	To show the mechanism of formation of lamellipodia in MCF10A cells
	Harris et al., 2014^62^	40 µM	1 hour	To assess the mechanism controlling establishment of tissue-level tension in MDCK-II cell monolayers
	Kajita et al., 2014^63^	25 µM	12-24 hours	To assess apical extrusion of ts-Src- or RasV12-MDCK cells
	Lechuga et al., 2014^64^	50 µM	24 hours	To assess (epithelial to myofibroblast transition) EMyT induction in A549 cells

**Table 2:** Ingenuity Pathway Analysis on early p53 response genes identified by Allen *et al.*, 2014^74^.

Categories	Diseases or Functions Annotation	p-Value	Molecules	# Molecules
Cellular Movement	cell movement	9,41E-07	ACKR2,ACTA2,ALOX5,APAF1,BAX,BTG2,CD82,CDKN1A,COL17A1,COL4A1,DDB2,DGKA,DOCK8,DRAM1,EBI3,EFNB1,FAS,FBXW7,GDF15,GPR56,ICAM1,INPP5D,ITGA3,ITGA9,KCNN4,KDM4B,LAMA3,LRP1,mir-34,NTF3,NTRK2,PML,PRDM1,PRKX,PTP4A1,PTPRU,RHOD,SDC1,SERPINB5,TP53INP1,TRAF4,UNC5B,VCAN	43
Cellular Movement	migration of cells	6,16E-06	ACKR2,ACTA2,ALOX5,APAF1,BAX,BTG2,CD82,CDKN1A,COL17A1,COL4A1,DOCK8,EBI3,EFNB1,FAS,FBXW7,GPR56,ICAM1,INPP5D,ITGA3,ITGA9,KDM4B,LAMA3,LRP1,mir-34,NTF3,NTRK2,PML,PRDM1,PRKX,PTP4A1,PTPRU,RHOD,SDC1,SERPINB5,TP53INP1,TRAF4,UNC5B,VCAN	38
Cellular Movement	invasion of breast cancer cell lines	1,83E-04	CD82,CDKN1A,DDB2,DGKA,FAS,ITGA3,LRP1,mir-34,SERPINB5	9
Cell-mediated Immune Response, Cellular Movement, Hematological System Development and Function, Immune Cell Trafficking	T cell migration	2,51E-04	ACKR2,ALOX5,COL4A1,DOCK8,FAS,ICAM1,INPP5D,ITGA3,ITGA9	9
Cellular Movement	invasion of tumor cell lines	3,29E-04	ACTA2,CD82,CDKN1A,DDB2,DGKA,DRAM1,FAS,GDF15,ITGA3,KDM4B,LRP1,mir-34,RHOD,SERPINB5,UNC5B,VCAN	16
Cellular Movement	invasion of cells	3,47E-04	ACTA2,CD82,CDKN1A,DDB2,DGKA,DRAM1,FAS,GDF15,ITGA3,KDM4B,LRP1,mir-34,NTRK2,RHOD,SDC1,SERPINB5,UNC5B,VCAN	18
Cellular Movement, Hematological System Development and Function, Immune Cell Trafficking	Lymphocyte migration	6,50E-04	ACKR2,ALOX5,COL4A1,DOCK8,EFNB1,FAS,ICAM1,INPP5D,ITGA3,ITGA9	10
Cellular Movement	cell movement of tumor cell lines	7,44E-04	ACTA2,CD82,CDKN1A,DDB2,DRAM1,EBI3,EFNB1,GDF15,KCNN4,KDM4B,LAMA3,LRP1,mir-34,NTF3,PTPRU,SDC1,SERPINB5,TP53INP1,TRAF4	19
Cell-mediated Immune Response, Cellular Movement, Hematological System Development and Function, Immune Cell Trafficking	cell movement of T lymphocytes	2,53E-03	ACKR2,COL4A1,DOCK8,FAS,ICAM1,INPP5D,ITGA3	7
Cellular Movement, Hematological System Development and Function, Immune Cell Trafficking	cell movement of mononuclear leukocytes	2,82E-03	ACKR2,ALOX5,COL4A1,DOCK8,EFNB1,FAS,ICAM1,INPP5D,ITGA3,ITGA9,UNC5B	11
Cellular Movement, Nervous System Development and Function	migration of neurons	2,95E-03	APAF1,BAX,COL4A1,EFNB1,GPR56,ITGA3,NTRK2	7
Cellular Movement, Renal and Urological System Development and Function	migration of kidney cell lines	3,90E-03	EFNB1,ICAM1,PRKX,PTP4A1	4
Cellular Movement, Hematological System Development and Function, Immune Cell Trafficking, Inflammatory Response	migration of phagocytes	4,99E-03	COL4A1,DOCK8,FAS,ICAM1,INPP5D,LRP1,SDC1	7
Cellular Movement, Hematological System Development and Function, Immune Cell Trafficking	migration of peripheral blood lymphocytes	6,34E-03	EFNB1,ICAM1	2
Cellular Movement, Hematological System Development and Function, Immune Cell Trafficking	cell movement of leukocytes	6,60E-03	ACKR2,ALOX5,CDKN1A,COL4A1,DOCK8,EFNB1,FAS,ICAM1,INPP5D,ITGA3,ITGA9,LRP1,PRDM1,SDC1,UNC5B	15
Cellular Movement	migration of epithelial cells	6,64E-03	CD82,COL17A1,ICAM1,LAMA3	4
Cellular Movement, Hematological System Development and Function	migration of hematopoietic progenitor cells	6,94E-03	DOCK8,ICAM1,ITGA3	3
Cell-mediated Immune Response, Cellular Movement, Hematological System Development and Function, Hematopoiesis, Immune Cell Trafficking	migration of thymocytes	7,27E-03	DOCK8,ITGA3	2
Cellular Movement	migration of tumor cell lines	8,25E-03	ACTA2,CD82,CDKN1A,EBI3,EFNB1,KDM4B,LAMA3,LRP1,mir-34,NTF3,PTPRU,SDC1,TP53INP1,TRAF4	14
Cellular Movement, Hematological System Development and Function, Immune Cell Trafficking, Inflammatory Response	migration of bone marrow-derived macrophages	8,26E-03	LRP1,SDC1	2
Cell-To-Cell Signaling and Interaction, Cell-mediated Immune Response, Cellular Movement, Hematological System Development and Function, Immune Cell Trafficking, Tissue Development	adhesion of regulatory T lymphocytes	8,66E-03	ICAM1	1

© 2000-2014 QIAGEN. All rights reserved.

**Table 3:** List of primers used for RT-qPCR.

*Gene*	Primer #1 (5'-3')	Primer #2 (5'-3')
Cyclophilin *(PPID)*	CATCTGCACTGCCAAGACTGA	TTGCCAAACACCACATGCTT
*GAPDH*	GCCTCAAGATCATCAGCAATGC	CCACGATACCAAAGTTGTCATGG
p53 *(TP53)*	AGGCCTTGGAACTCAAGGAT	CCCTTTTTGGACTTCAGGTG
*DAAM1*	GGAGCTACAAGTTGGCCTGA	TCCTTCTCTAAAGCCAGCAGA
*DAAM2*	CAAAGCCCAAAGTGGAAGC	CATCTGTCTAAGACGCTTGCTG
*FHOD1*	AGTCTCGTGCCAAAGAGGTG	TCCAGCACTGTGGTCATTGT
*FHOD3*	AGGCCAGGTTGGAAAGGT	TCTGCTGCCAGTGACTCTTG
*FMNL1*	CTACGCGCCATCATGAACT	ACACAGGCTGGGTGGTTC
*FMNL2*	TGTGGAACTGGAAAAGCAACT	TGTGTGAACTTGAGTATTTGCATC
*FMNL3*	CCATCGAGGACATCATCACA	CCGAGAGGGTCTCAGTGG
*INF1*	GCATCATGTTCAGAAGACTGCTA	TGTCCTGACAAACAGCAAGTG
*INF2*	GAGGTCTTTGCCTCCCTGTT	GACAGGAGCTGGGCAGAC
*DELPHILLIN*	AAGAGTTCAGCCGCAAGG	TGCTCAGCTGCAAACTGC
*FMN1*	GGCCCCTCTGATTCCAAA	GCTTGAAGTCTGCCAGGAGT
*FMN2*	GCTTCCAGAACGTGTTCACAG	ATCCGGGAGCAAAACTTCTC
mDia1 *(DIAPH1)*	TTGGACATTCTTAAACGACTTCAT	GCTTGTTCCGGCTATCGTAA
mDia2 *(DIAPH3)*	GCGGGAAAAGGACTTCAGTAT	TCTGTCGGCTTCTCTTAAGACTTC
mDia3 *(DIAPH2)*	TGCATTTTGAGAAGAACAAAGTG	CCAGCTTATCTTGATCTTTGCAG
