# Supplementary material for: SMIFH2 has effects on Formins and p53 that perturb the cell cytoskeleton
Source: Sci Rep. 2015 Apr 30;5:9802. doi: 10.1038/srep09802 (PMC5386218; doi:10.1038/srep09802)
Supplement: Supplementary Information [file srep09802-s1.pdf]

SMIFH2 has effects on Formins and p53 that perturb the cell cytoskeleton.

Tadamoto Isogai, Rob van der Kammen, and Metello Innocenti

Division of Molecular Genetics, The Netherlands Cancer Institute, Amsterdam, The Netherlands

Correspondence and request for materials should be addressed to M.I. (m.innocenti@nki.nl)

Supplementary Information

Supplementary Figures

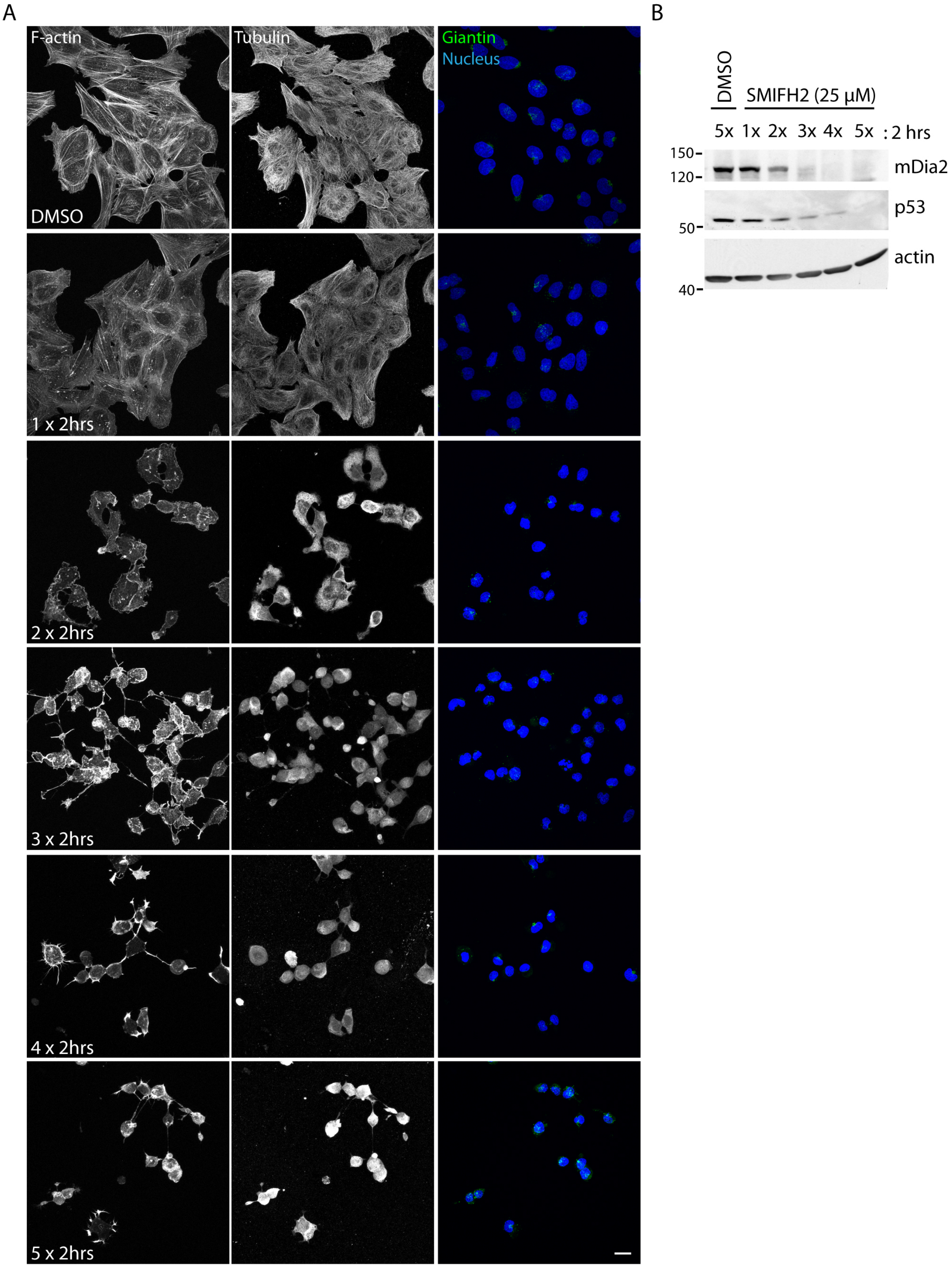

Figure S1 | SMIFH2 undergoes intracellular breakdown and/or inactivation.

(A) Repeated addition of fresh SMIFH2 causes a progressive and persistent depolymerization of both F-actin and microtubules results. U2OS cells were treated with either SMIFH2 or DMSO and, every two hours, the medium was replaced with either new SMIFH2- or new DMSO-containing medium. The number of two-hour cycles (from 1 to 5) is indicated as 1-5 x 2hrs. Media were prepared at the beginning of the time course. Fixed cells were stained with anti-β-tubulin (Alexa-488), TRITC-conjugated phalloidin to visualize F-actin, anti-Giantin antibodies (Alexa-647; green in merge) and DAPI to stain the nucleus (blue in merge). Representative maximal confocal projections are shown. Scale bar, 20 μm. (B) Repeated addition of fresh SMIFH2 causes a progressive and persistent downregulation of p53. Parallel samples of U2OS cells were treated with either DMSO or SMIFH2 as in (A). Total cell lysates were separated by SDS-PAGE and blotted with the indicated antibodies. Actin served as loading control. Gels were run under the same experimental conditions and blots were cropped for final display.

A

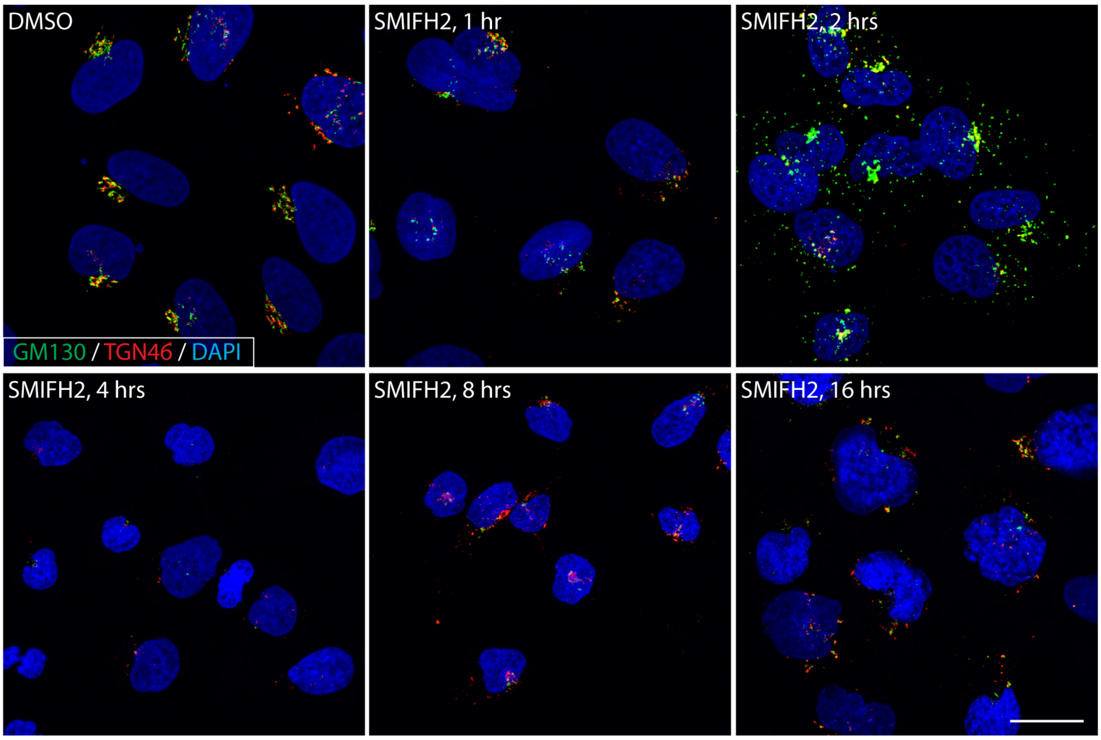

Figure S2 | SMIFH2 disperses the Golgi network.

(A) U2OS cells were treated with SMIFH2 or DMSO for the indicated time (hr = hour) as in Fig. 1A. Fixed cells were stained with anti-GM130 (Alexa-488; green in merge) and anti-TGN46 antibodies (Alexa-647; red in merge). Nuclei were stained with DAPI. Maximal confocal projections are shown. Scale bar, 20 μm.

A

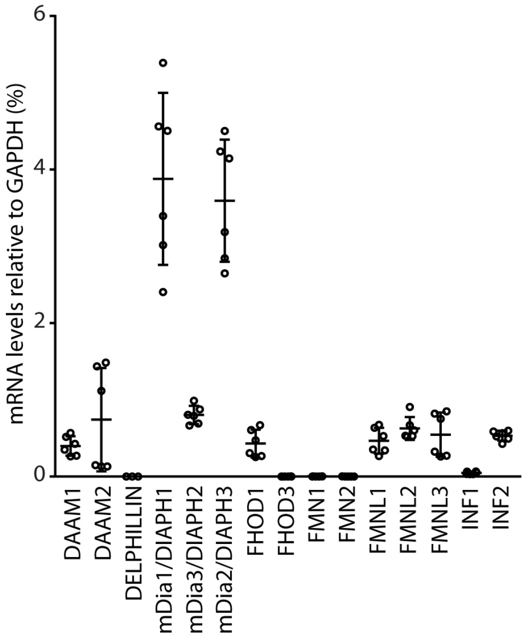
mRNA levels relative to GAPDH (%)
0
2
4
6
DAAM1
DAAM2
DELPHILLIN
mDia1/DIAPH1
mDia3/DIAPH2
mDia2/DIAPH3
FHOD1
FHOD3
FMN1
FMN2
FMNL1
FMNL2
FMNL3
INF1
INF2

B

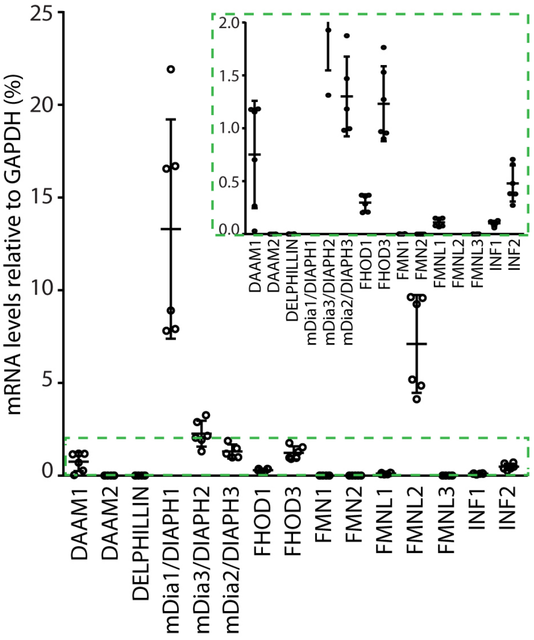
mRNA levels relative to GAPDH (%)
0
5
10
15
20
25
0.0
0.5
1.0
1.5
2.0
DAAM1
DAAM2
DELPHILLIN
mDia1/DIAPH1
mDia3/DIAPH2
mDia2/DIAPH3
FHOD1
FHOD3
FMN1
FMN2
FMNL1
FMNL2
FMNL3
INF1
INF2

C

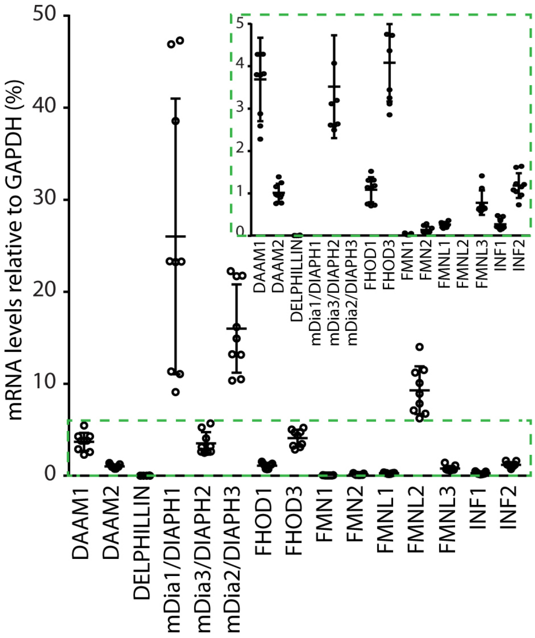
mRNA levels relative to GAPDH (%)
0
10
20
30
40
50
0
1
2
3
4
5
DAAM1
DAAM2
DELPHILLIN
mDia1/DIAPH1
mDia3/DIAPH2
mDia2/DIAPH3
FHOD1
FHOD3
FMN1
FMN2
FMNL1
FMNL2
FMNL3
INF1
INF2

Figure S3 | U2OS, HCT116 and 293T cells have distinct Formin expression profiles.

(A-C) Formin expression profile of U2OS, HCT116 and 293T cells. Expression of Formins was assessed by RT-qPCR starting from total mRNA isolated from (A) U2OS, (B) HCT116 and (C) 293T cells. Green dashed boxes enclose blown-up area. Data are presented as means ± s.d. of at least two independent experiments, each consisting of three technical replicates.

A

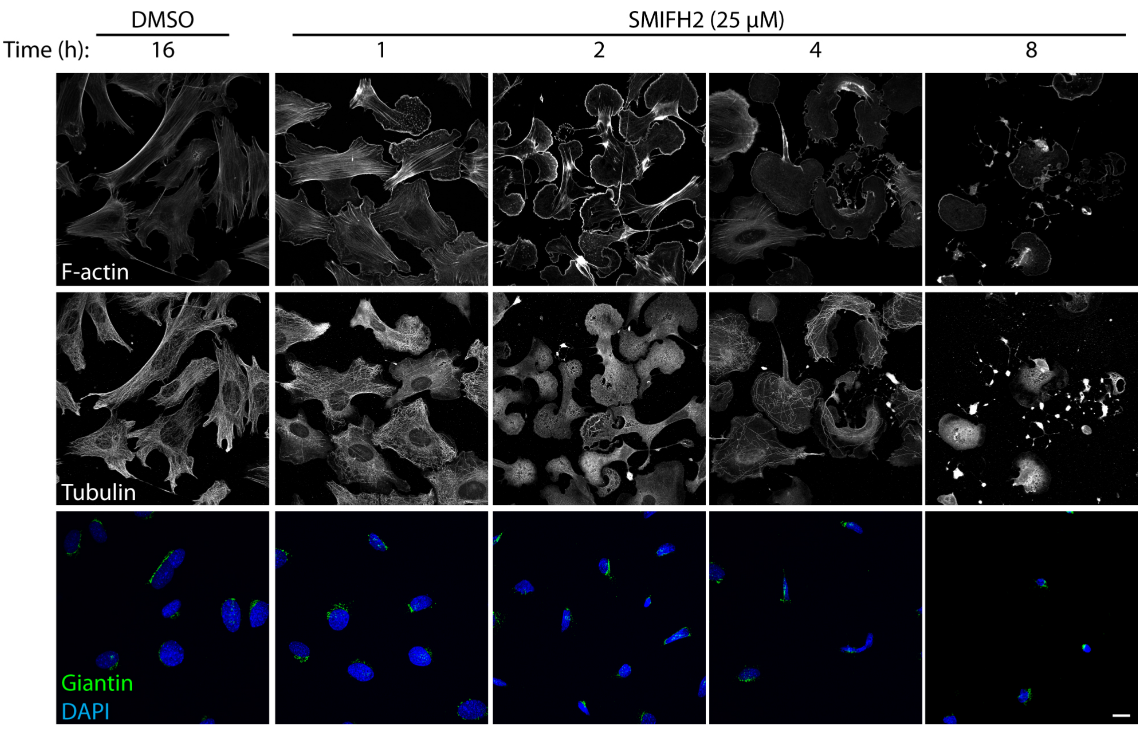

B

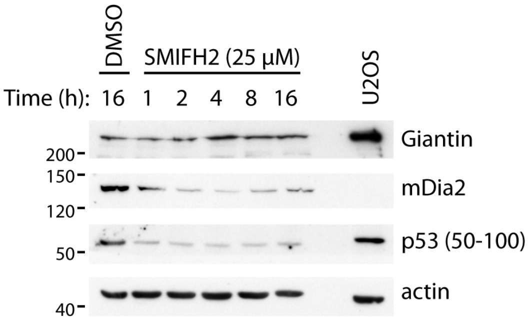

Figure S4 | SMIFH2 affects the cytoskeleton, but not the Golgi complex of MEF cells.

(A) SMIFH2 induces dynamic cytoskeletal remodelling in MEF cells. MEF cells were treated with SMIFH2 or DMSO for the indicated time (Time, (h) = hour). Fixed cells were stained with anti-β-tubulin (Alexa-488) and anti-Giantin antibodies (Alexa-647; green in merge), TRITC-conjugated phalloidin to visualize F-actin, and DAPI to stain the nucleus (blue in merge). Representative maximal confocal projections are shown. Scale bar, 20 µm. (B) Downregulation of mDia2 and p53 by SMIFH2 temporally overlap. MEF cells were treated with either DMSO or SMIFH2 in parallel as in (A). Total cell lysates were separated by SDS-PAGE and blotted with the indicated antibodies. Actin served as loading control. Gels were run under the same experimental conditions and blots were cropped for final display.

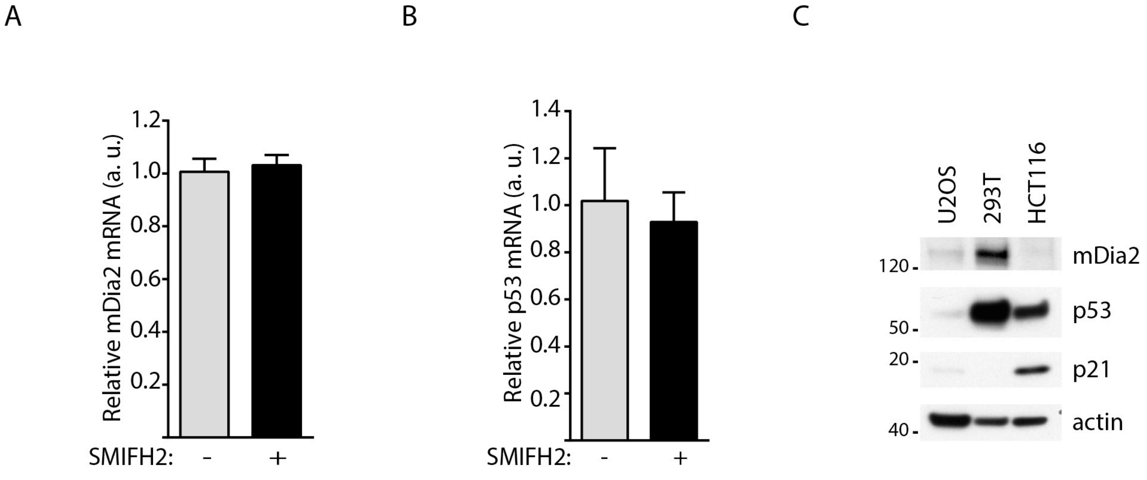

Figure S5 | SMIFH2 does not alter p53 and mDia2 messenger levels and comparison of p53 levels in U2OS, 293T and HCT116 cells.

(A-B) SMIFH2 does not alter the relative expression of the p53 and mDia2 messenger in 293T cells. 293T cells were treated as in Fig. 3A. After isolating total RNA and cDNA synthesis, qPCR analyses were performed using *mDia2* or *p53*-specific primers. *Cyclophilin* was used as a normalizing gene. Relative mRNA levels of (A) *mDia2* and (B) *p53* were measured in control DMSO-treated (-) and SMIFH2-treated (+) cells. The values measured in the DMSO-treated samples served as a reference. Data are presented as means ± s.d. of two independent experiments, each consisting of three technical replicates. The downregulation of both mDia2 and p53 protein levels in the SMIFH2-treated cells was confirmed by immunoblotting using lysates obtained from parallel samples presented in Fig. 4A. (C) Characterization of mDia2, p53 and p21 levels in U2OS, 293T and HCT116 cell lines. Total cell lysates were separated by SDS-PAGE and blotted for the indicated antibodies.

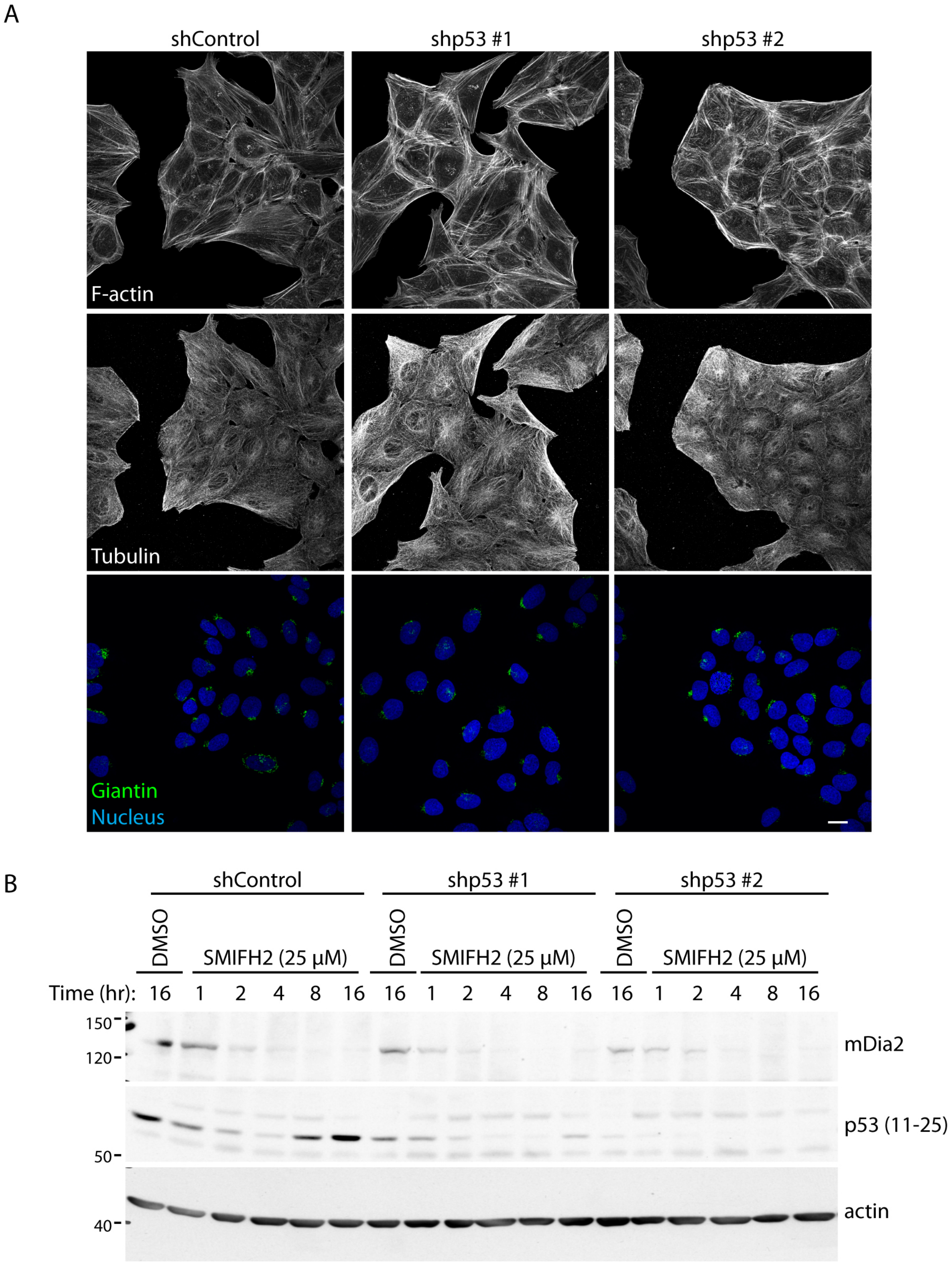
A
shControl
shp53 #1
shp53 #2
F-actin
Tubulin
Giantin
Nucleus
B
shControl
shp53 #1
shp53 #2
DMSO
SMIFH2 (25 μM)
Time (hr): 16 1 2 4 8 16 16 1 2 4 8 16 16 1 2 4 8 16
150
120
50
40
mDia2
p53 (11-25)
actin

Figure S6 | p53 levels affects the actin cytoskeleton in U2OS cells.

(A) Cortical actin cytoskeleton and the stress fibres are more prominent in p53 knockdown U2OS cells. Growing control (shControl) and p53 knockdown (shp53 #1 and #2) U2OS cells were fixed and stained with TRITC-conjugated phalloidin to visualize F-actin, anti-β-tubulin (Alexa-488), anti-Giantin antibodies (Alexa-647; green in merge) and DAPI to stain the nucleus (blue in merge). Representative maximal confocal projections are shown. Scale bar, 20 µm. (B) Downregulation of mDia2 by SMIFH2 is independent of p53. U2OS cells were treated with either DMSO or SMIFH2 for the indicated time (hr = hour). Total cell lysates were separated by SDS-PAGE and blotted with the indicated antibodies. Actin served as loading control. Gels were run under the same experimental conditions and blots were cropped for final display.

Supplementary Movies

Movie S1 | SMIFH2 induces dynamic remodeling of the actin cytoskeleton and microtubules.

U2OS cells expressing EGFP-LifeAct and mCherry-α-Tubulin were plated on gelatin-coated transwell plates and imaged as described in the Methods. A representative video of one out of two experiments showing similar results is displayed. Scale bar, 20 μm.

Movie S2 | SMIFH2-treated cells show are transient increase in migration.

U2OS cells were plated on gelatin-coated multiwell plates and imaged as described in the Methods. Cells entering mitosis are indicated with arrows. A representative video of one out of two experiments showing similar results is displayed. Scale bar, 40 μm.

Movie S3 | High concentration of SMIFH2 triggers cell death independently of p53.

U2OS, 293T, HCT116 wt and p53-/- cells were plated on gelatin-coated multiwell plates and treated with 50 μM of SMIFH2 and imaged as described in the Methods. A representative video of one out of two experiments showing similar results is displayed.

Movie S4 | Dynamics of actin cytoskeleton in control and p53 knockdown U2OS cells treated with SMIFH2.

Control and p53 knockdown (#1 and #2) U2OS cells expressing EGFP-LifeAct and mCherry-α-Tubulin were plated on gelatin-coated glass-bottom dishes and imaged as described in the Methods. A representative video of one out of two experiments is displayed. p53 knockdown cells were imaged in parallel with the control cells from Movie S1 using identical acquisition settings. Note that lamellipodial protrusions are absent in both p53 knockdown cell lines. Scale bar, 20 μm.

Movie S5 | Dynamics of microtubules in control and p53 knockdown U2OS cells treated with SMIFH2.

Control and p53 knockdown (#1 and #2) U2OS cells expressing EGFP-LifeAct and mCherry-α-Tubulin were plated on gelatin-coated transwell plates and imaged as described in the Methods. A representative video of one out of two experiments is displayed. Note that these are the same cells imaged in Movie S4. Scale bar, 20 μm.

Movie S6 | Dynamics of microtubules in control and p53 knockdown U2OS cells treated with SMIFH2.

Control and p53 knockdown (#2) U2OS cells expressing EGFP-LifeAct and ECFP-Golgi were plated on gelatin-coated transwell plates and imaged as described in the Methods. A representative video of one out of two experiments showing similar results is displayed. All cells were acquired in parallel with the same settings. Scale bar, 20 μm.
